# Genomic Characterization of *mcr-1.1*-Producing *Escherichia coli* Recovered From Human Infections in São Paulo, Brazil

**DOI:** 10.3389/fmicb.2021.663414

**Published:** 2021-06-09

**Authors:** Raquel Girardello, Carlos Morais Piroupo, Joaquim Martins, Marcia Helena Maffucci, Ana Paula Cury, Maria Renata Gomes Franco, Fernanda de Mello Malta, Natália Conceição Rocha, João Renato Rebello Pinho, Flavia Rossi, Alberto José da Silva Duarte, João Carlos Setubal

**Affiliations:** ^1^Laboratório de Microbiologia Molecular e Clínica, Programa de Pós-Graduação em Ciências da Saúde, Universidade São Francisco, Braganca Paulista, Brazil; ^2^Departamento de Bioquímica, Instituto de Química, Universidade de São Paulo, São Paulo, Brazil; ^3^Hospital das Clínicas, Divisão Laboratório Central, Faculdade de Medicina, Universidade de São Paulo, São Paulo, Brazil; ^4^Laboratório de Técnicas Especiais, Hospital Israelita Albert Einstein, São Paulo, Brazil

**Keywords:** Polymyxin, colistin, plasmid, insertion sequence, hospital dissemination

## Abstract

Polymyxins are one of most important antibiotics available for multidrug-resistant Gram-negative infections. Diverse chromosomal resistance mechanisms have been described, but the polymyxin resistance phenotype is not yet completely understood. The objective of this study was to characterize colistin resistant *mcr-1*-producing strains isolated from human infections over one year in a hospital setting (Hospital das Clínicas, São Paulo, Brazil). We isolated 490 colistin-resistant Gram-negative rods, of which eight were *mcr-1.1*-positive *Escherichia coli*, the only species with this result, indicating a low incidence of the *mcr-1* production mechanism among colistin-resistant isolates. All *mcr-1.1* positive isolates showed similarly low MICs for colistin and were susceptible to most antibiotics tested. The isolates showed diversity of MLST classification. The eight *mcr-1.1*-positive *E. coli* genomes were sequenced. In seven of eight isolates the *mcr-1.1* gene is located in a contig that is presumed to be a part of an IncX4 plasmid; in one isolate, it is located in a contig that is presumed to be part of an IncHI2A plasmid. Three different genomic contexts for *mcr-1.1* were observed, including a genomic cassette *mcr-1.1-pap2* disrupting a DUF2806 domain-containing gene in six isolates. In addition, an IS1-family transposase was found inserted next to the *mcr-1.1* cassette in one isolate. An *mcr-1.1-pap2* genomic cassette not disrupting any gene was identified in another isolate. Our results suggest that plasmid dissemination of hospital-resident strains took place during the study period and highlight the need for continued genomic surveillance.

## Introduction

Multidrug-resistant Gram-negative bacterial clinical isolates are responsible for high mortality rates worldwide (Agência Nacional de Vigilância Sanitária, 2016; De Waele et al., 2020), and constitute a challenge for healthcare professionals with respect to therapy choice, because few options are currently available. Isolates that are resistant to antibiotics that have been recently produced by the pharmaceutical industry have already been reported (Wang et al., 2020). Moreover, these new antibiotics have no activity against carbapenem-resistant *Acinetobacter baumannii* (Zhanel et al., 2013; Nelson et al., 2017; Shields et al., 2017; Rodriguez et al., 2018; Noval et al., 2020). Thus, polymyxins remain the antimicrobial of last resort for treatment of infections caused by these microorganisms.

The increase of clinical use of polymyxins has been associated with elevation of the resistance rates for antibiotics of this class (Lee et al., 2015; Girardello et al., 2017; Rossi et al., 2017a; Braun et al., 2018; Zavascki et al., 2018). Briefly, the molecular mechanism of polymyxins resistance involves two component systems, which regulate modifications in the bacterial membranes, reducing the negative charge of bacterial surface, and consequently, decreasing the interaction between polymyxins and bacterial cell (Moffatt et al., 2019).

In 2015, the plasmidial mechanism of colistin resistance *mcr-1* was described by Liu et al. (2016). This report left the clinical community alarmed, due to the possibility of further dissemination of this kind of resistance. The mcr-1 mechanism acts by adding phosphoethanolamine in the lipid A of the outer membrane lipopolysaccharide, decreasing the negative charge of the bacterial surface (Gao et al., 2016). In follow-up studies, *mcr* genes have been found in many other isolates, in different parts of the world (Li et al., 2016; García et al., 2018). After the first description of this gene, several studies have shown that it was also present in strains isolated over three decades ago (Arcilla et al., 2016; Hu et al., 2016; Kluytmans-van den Bergh et al., 2016; Olaitan et al., 2016; Suzuki et al., 2016; Tse and Yuen, 2016; Elbediwi et al., 2019).

In 2016, *mcr-1* was detected in our institution for the first time, in a colistin-resistant *Escherichia coli* isolate from blood culture site (Rossi et al., 2017b). The present study aimed to evaluate the further dissemination in our institution of resistance to colistin caused by the *mcr* plasmid-located gene after the 2016 detection, and to characterize de genome of the *mcr* producing isolates.

## Materials and Methods

### Colistin Resistant Isolates

All colistin resistant Gram-negative strains, isolated between April, 2016 and March, 2017 in the Hospital das Clínicas, a hospital complex of São Paulo, Brazil, were screened by PCR, for the presence of *mcr* genes, using specific primers, previously described (Liu et al., 2016, 2020; Rebelo et al., 2018). The isolates identification was performed using Vitek MS (bioMeriéux, France).

### Antimicrobial Susceptibility Tests

The antimicrobial susceptibility test was performed by using Vitek-2 (bioMeriéux, France), and the colistin resistance was prospectively confirmed using CLSI broth microdilution (CLSI, 2020).

### Whole Genome Sequencing

The *mcr-1* positive Gram-negative isolates were submitted to whole genome sequencing using the MiSeq Platform (Illumina, Inc., United States), after DNA extraction using QiaAmp DNA Mini Kit (Qiagen, Germany). The DNA quantification was performed using QubitTM dsDNA HS Assay Kit, according to manufacture recommendations (ThermoFisher, MA, United States). The sequencing libraries were prepared from 1 ng of total DNA using the Illumina Nextera XT DNA library preparation kit (Illumina, Inc., United States), according to the manufacture recommendations, and the sequencing run was performed in the Illumina MiSeq to generate 250 bp paired end reads.

### Bioinformatics

Genome assembly was performed using A5-miseq (Coil et al., 2015), ABySS 2.2.4 (Jackman et al., 2017), DISCOVAR (Weisenfeld et al., 2014), MaSuRCA 3.3.3 (Zimin et al., 2013), MIRA 4.9.6 (Chevreux et al., 1999), and SPAdes 3.13.1 (Bankevich et al., 2012). These assemblies were then combined into a single superior assembly using Metassembler 1.5 (Wences and Schatz, 2015). The merging of Metassembler is iterative, using the locally best sequences of the next assembly to improve the last, so it was convenient to rank the six assemblies by N50 beforehand. The next step was a scaffolding with MeDuSa 1.6 (Bosi et al., 2015) using five of the closest genomes found on NCBI as reference. The all-against-all genome comparison was done with fastANI (Jain et al., 2018). The phylogenetic tree was inferred using the program parsnp (Treangen et al., 2014).

Multi-locus sequence typing was done using the server available at https://pubmlst.org. Identification of plasmids was done with PlasmidFinder 2.0.1 (Carattoli et al., 2014), and antibiotic resistance genes were found with ResFinder 3.2 (Zankari et al., 2012).

## Results

### Colistin Resistant Clinical Isolates

During one year of investigation, 7,852 Gram-negative bacilli were isolated in the hospital complex; 490 (6.24%) Gram-negative bacilli were resistant to colistin, of which 399 were classified as *Klebsiella pneumoniae* (81.43%), 29 as *A. baumannii* complex (5.92%), 19 as *E. coli* (3.88%), 19 as *Enterobacter cloacae* (3.88%), 18 as *Pseudomonas aeruginosa* (3.67%), 3 as *Citrobacter freundii* (0.61%), 2 as *Klebsiella aerogenes* (0.41%), and 1 as *Citrobacter youngae* (0.20%). Among the 490 colistin-resistant isolates, 418 (85.3%) showed colistin Minimal Inhibitory Concentration (MIC) > 16 μg/mL, and 72 (14.7%) showed borderline MICs, from 4 to 8 μg/mL.

### *mcr-1*-Producing Isolates

All 490 colistin-resistant isolates were submitted to PCR for *mcr* gene screening, and *mcr-1* was detected in eight *E. coli* isolates, corresponding to 1.63% of all colistin resistant Gram-negative bacilli, and to 42% of all *E. coli* isolates recovered in the hospital during the study. *mcr-1* was not detected in any other colistin-resistant species. None of the *mcr-2* through *mcr-10* variants were detected in the examined strains. The *mcr-1*-positive strains were recovered from urine (three isolates), blood culture (two isolates), and bone, soft-tissue, and peritoneal fluid (one isolate, each). The antimicrobial susceptibility profile from eight isolates is described in Table 1. All *mcr-1*-producing isolates showed similar antimicrobial susceptibility profiles, being susceptible to third-generation cephalosporins and carbapenems antibiotics and showing borderline colistin MICs (4 and 8 μg/mL, four isolates each). Five of eight *mcr-1*-producing *E. coli* isolates were simultaneously resistant to ciprofloxacin. Three strains (Ec482, Ec483, and Ec716) were isolated from the same patient (Patient 2; bone, soft tissue, and urine, respectively) (Table 2). All patients from whom the *mcr-1*-producing strains were isolated had their infections successfully treated.

**TABLE 1:**
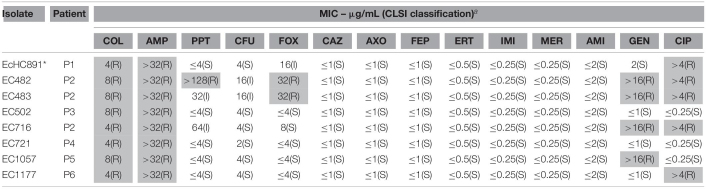
Antimicrobial susceptibility profile of *mcr-1-*producing *E. coli* isolated during one year.

^a^Break-point interpretation according to CLSI (2020). *The clinical case of isolate EcHC891 has been previously reported (Rossi et al., 2017b). COL, colistin; AMP, ampicillin; PPT, piperacillin/tazobactam; CFU, cefuroxime; FOX, cefoxitin; CAZ, ceftazidime; AXO, ceftriaxone; FEP, cefepime; ERT, ertapenem; IMI, imipenem; MER, meropenem; AMI, amikacin; GEN, gentamicin; CIP, ciprofloxacin. S, susceptible; I, intermediate; R, resistant. The highlighted MICs correspond to resistance category.

**TABLE 2:** Characterization of *mcr-1-*producing *E. coli* isolates.

**Isolate**	**Patient**	**Infection Site**	**Date**	**MLST**^*a*^	***mcr-1.1* contig**^*b*^	**Genomic context of *mcr-1.1***^*c*^	**Antibiotic resistance genes**^*d*^
EcHC891*	P1	Blood Culture	Apr/2016	ST 156	IncX4 (33.2 Kb)	I	*mcr-1.1; aac(6’)Ib-cr; bla*_*TEM*__1_
Ec482	P2	Bone	Aug/2016	ST 410	IncX4 (72.1 Kb)	I	*mcr-1.1; aac(3)-iib; aph(3”)-ib; aph(6)-id; floR; sul2; bla*_*TEM*__1_
Ec483	P2	Soft Tissue	Aug/2016	ST 410	IncX4 (32.7 Kb)	I	*mcr-1.1; aac(3)-iib; aph(3”)-ib; aph(6)-id; floR; sul2; bla*_*TEM*__1_
Ec502	P3	Urine	Aug/2016	ST 648	IncHI2A (63.2 Kb)	III	*mcr-1.1; aadA; aph(3”)-ib; aph(6)-id; sul1; bla*_*TEM*__1_
Ec716	P2	Urine	Oct/2016	ST 410	IncX4 (9.8 Kb)	I	*mcr-1.1; aac(3)-iib; aph(3”)-ib; aph(6)-id; floR; sul2; bla*_*TEM*__1_
Ec721	P4	Peritoneal Fluid	Oct/2016	ST 10	IncX4 (42.1 Kb)	II	*mcr-1.1; aph(3”)-ib; aph(6)-id; sul2; bla*_*TEM*__1_
Ec1057	P5	Urine	Jan/2017	ST 10	IncX4 (9.6 Kb)	I	*mcr-1.1; aac(3)-iib; aadA3; aph(3”)-ib; aph(6)-id; floR; sul2; bla*_*TEM*__1_
Ec1177	P6	Blood Culture	Mar/2017	ST 744	IncX4 (9.8 Kb)	I	*mcr-1.1; aadA; aadA2; aph(3”)-ib; aph(3’)-ia; aph(6)-id; qacH; sul2; sul3; bla*_*TEM*__1_

^a^MLST determined by https://pubmlst.org/. ^b^These are the mcr-1.1-contaning contigs, and these contigs contained the indicated plasmid markers as given by PlasmidFinder 2.0.1 (Carattoli et al., 2014). ^c^The mcr-1.1 locus tag is described in Table 3. ^d^The antibiotic resistance genes were found with ResFinder 3.2 (Zankari et al., 2012). *The clinical case of isolate EcHC891was previously reported (Rossi et al., 2017b). For this work, all genomic analyses described in section “Materials and Methods” were redone for this genome.

### Whole Genome Sequencing

**FIGURE 1:**
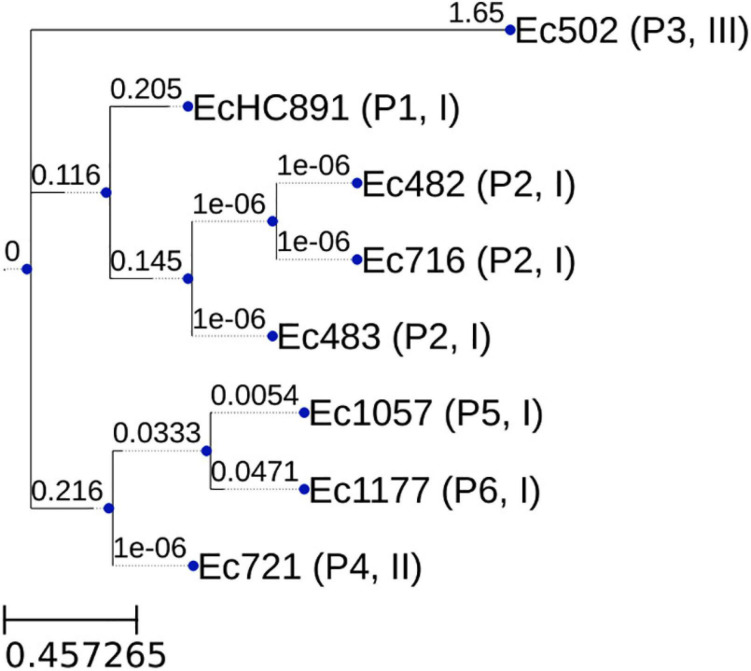
Phylogenetic tree generated by program parsnp (core genome SNP tree). The tree was inferred with FastTree 2.1 (Price et al., 2009). The numbers above branches are lengths, measured in number of substitutions per site. Leaf labels: Isolate identification (Patient number, Genomic Context type). The core genome size was 3,812,930 bp. This resulted from multiplying the individual genome cluster coverage value by its size, adding up these results, and dividing by the total number of genomes in the core genome computation.

We have sequenced the genomes of the eight *mcr-1*-positive *E. coli* isolates; genome sequencing data and accession numbers are given in Table 3; all genomes are in draft status. Genome size varies from 4,758,738 to 5,770,437 bp. CheckM (Parks et al., 2015) results showed that all genomes had at least 99.04% completeness and at most 0.55% contamination. The genome of isolate EcHC891 has been reported previously (Rossi et al., 2017b) and is included here because it was isolated during the same period as the others. The isolates showed diversity of sequence types (STs): ST156 (Patient 1); ST410 (3 isolates from Patient 2); ST648 (Patient 3), ST10 (Patient 4 and Patient 5), and ST744 (Patient 6) (Table 2). We also performed a whole genome comparison of all against all (Supplementary Table 1) and inferred a phylogenetic tree from single nucleotide polymorphisms (Figure 1).

**TABLE 3:** Whole genome sequencing data.

**Isolate**	**#scaffolds/contigs**	**Genome Size (bp)**	***mcr-1.1* locus tag**	**GenBank Accession Number**
EcHC891*	70	5,770,437	BSF34_26145	MRDN01000000
Ec482	11	4,977,896	FQP73_14800	VNJD00000000
Ec483	12	4,960,890	FQP74_06700	VNJC00000000
Ec502	17	5,425,402	FQP78_17265	VNJB00000000
Ec716	8	4,970,368	FQP71_02855	VNJA00000000
Ec721	13	4,971,516	FQP75_17750	VNIZ00000000
Ec1057	18	4,887,624	FQP69_13845	VNIY00000000
Ec1177	9	4,758,738	FQP57_08120	VNIX00000000

*The case report of isolate EcHC891 has been previously published (Rossi et al., 2017b).

We found the *mcr-1.1* gene in each genome (Table 2). In seven isolates, *mcr-1.1* is located in a contig that is presumed to be part of an IncX4 plasmid (EcHC891, Ec482, Ec483, Ec716, Ec721, Ec1057, and Ec1177). In the Ec502 isolate, *mcr-1.1* is located in a contig that is presumed to be part of an IncHI2A plasmid (Table 2).

**FIGURE 2:**
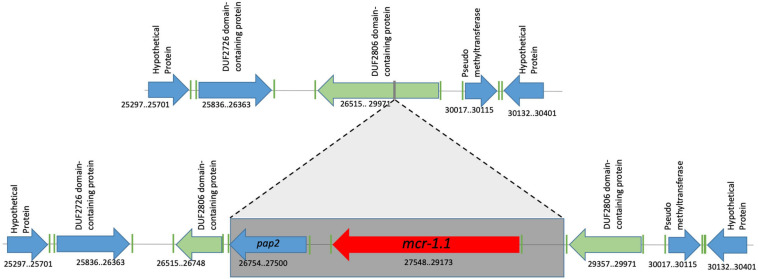
Genomic context #1 identified in EcHC891, Ec482, Ec483, Ec716, Ec1057 and Ec1177 isolates. The genomic cassette *mcr-1.1-pap2* was found disrupting a pre-existing DUF2806-domain-containing gene, without any associated insertion sequence. Genomic coordinates refer to scaffold 3 of the Ec482 genome.

We define the genomic context of the *mcr-1.1-pap2* cassette as including the flanking upstream and flanking downstream regions of lengths between 1000 and 2000 kbp (these lengths vary according to isolate, as will be seen). The genome characterization of the eight *mcr-1.1*-producing *E. coli* shows three different genomic contexts. The first group, including EcHC891, Ec482, Ec483, Ec716, Ec1057, and Ec1177 isolates, shows genomic context I. For these isolates, the genomic cassette *mcr-1.1-pap2* disrupts a pre-existing DUF2806-domain-containing gene (Figure 2). This genomic cassette has been described in diverse studies from samples of retail poultry meats and chicken meat from Korea, Japan, and Brazil (Accession numbers: MK875286.1; LC227558). In addition, this genomic context has also been identified in isolates from animals, such as migratory *Magellanic penguins*, and in human and chicken gut microbiota (Accession numbers: CP021419.1; CP017246.1, KY689633, and KY689632) (Donà et al., 2017; Sellera et al., 2017; Kim et al., 2020). The nucleotide sequence identity between the *mcr-1.1*-containing regions in all genomes of genomic context I is 100%.

**FIGURE 3:**
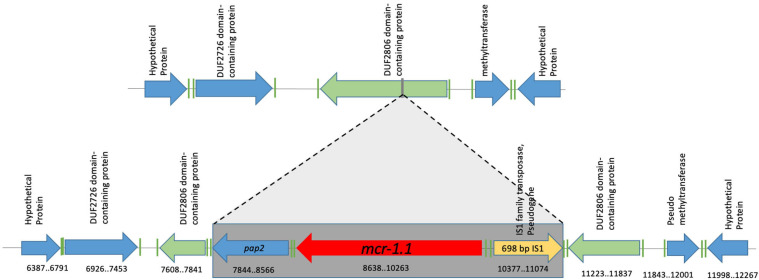
Genomic context #2 identified in the Ec721 isolate. A 698 bp Family 1 transposase was found upstream of the *mcr-1.1* gene, on the opposite strand; otherwise, the genomic context is the same as the one shown in Figure 2. Genomic coordinates refer to scaffold 2 of the Ec721 genome.

Genomic context II is represented by the Ec721 isolate and is identical to genomic context I, except for the presence of a 698-bp IS1-family transposase (which contains a frameshift, and has therefore been annotated as a pseudogene) inserted upstream of the *mcr-1.1* gene on the opposite strand (Figure 3). The *mcr-1.1* containing region in the Ec721 genome also has 100% nucleotide identity to the corresponding regions of context I genomes, excepting the IS1 transposase insertion.

**FIGURE 4:**
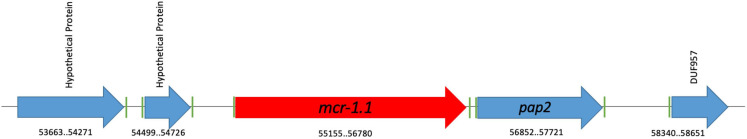
Genomic context #3 identified in the Ec502 isolate. Genomic coordinates refer to contig Qabyss.discovar.masurca.spades.a5_22 of the Ec502 genome.

Finally, genomic context III is represented by the Ec502 isolate. The genomic cassette *mcr-1.1-pap-2* was also found, but not disrupting a pre-existing gene. For this isolate we did not identify any IS in the genomic cassette (Figure 4). This same context has been described in accession numbers CP026933.2 and CP022165.1 (Gilrane et al., 2017). It is worth noting here that, besides being the only strain to present this *mcr-1* cassette composition, the genome of the Ec502 isolate is the most different from the others we sequenced (Supplementary Table 1).

In seven genomes (EcHC891, Ec482, Ec483, Ec716, Ec721, Ec1057, and Ec1177 isolates) the *pap-2* protein product has 247 aa, whereas in Ec502 it has 297 aa. BLAST alignments showed a GenBank record of this same protein product (EFD4953355.1) with 327 aa. The different lengths occur at the 3’ end of the protein sequence, and are therefore not due to different choices of the translation start codon. Based solely on genomic information (and given that the nucleotide similarity is very high between these *E. coli* strains) it is not possible to determine which of these lengths is the “correct” one: the shorter ones may have had a mutation that created a premature stop with respect to the longer one; or the longer ones could have had mutations that changed the stop codon to a non-stop codon; or all these variants may be different but nevertheless functional (i.e., these are not pseudogenes). The question seems to require experimental data to be clarified.

## Discussion

Colistin became the first-line drug for Gram-negative infections in patients admitted to the ICU in our institution in the last years, since the carbapenem resistance rates among *Acinetobacter* spp. increased from 30 to 70%, between 2010 and 2014 (Rossi et al., 2017a). The polymyxins resistance can be induced during the therapy with the drug, so, despite the polymyxin susceptibility still showing relatively high rates for most bacterial species, the resistance rates have been increasing worldwide in the last few years (Braun et al., 2018; Zavascki et al., 2018; de Lastours et al., 2020).

It has been reported that most colistin resistant isolates presenting the *mcr-1* mechanism have polymyxin MICs around the breakpoint for resistance (Principe et al., 2018; Lee et al., 2019). In some cases, these genes are found among susceptible isolates (Fernandes et al., 2016; Aires et al., 2017). In this study, we evaluated clinical isolates with MICs classifying them as resistant, according to CLSI guidelines (≥4 μg/mL); and also found this gene among isolates with borderline MICs (4 and 8 μg/mL); however, differently from what was reported in a multicentric study (Principe et al., 2018), among all of the 11 examined *mcr-1*-negative colistin-resistant *E. coli*, we observed colistin MIC > 16 μg/mL (Supplementary Table 2). Because of this observation, in our institution, β-lactam susceptibility plus colistin resistance or borderline MIC values are phenotypes that trigger *mcr-1* screening.

After the first detection of *mcr-1* in our hospital in 2016 (Rossi et al., 2017b), this mechanism was sporadically detected. The *mcr-1* plasmid resistance mechanism appears to be less clinically important when compared to other mechanisms in our institution. In this study, the most common colistin-resistant species recovered was *K. pneumoniae*, representing more than 80% of all colistin-resistant isolates. Differently from what was observed among *E. coli* isolates, *K. pneumoniae* showed high resistant rates to all antimicrobials tested and elevated MICs for colistin (>16 μg/mL); however, *mcr-1* was not detected in these isolates (Supplementary File 3). The main colistin resistance mechanism among *K. pneumoniae* in our hospital appears to be the *mgrB* mutation (data not shown), as already reported in the literature (Cannatelli et al., 2014). On the other hand, *mcr-1* appears to be an important colistin resistance mechanism in *E. coli* strains.

Interestingly, in our study we isolated three *mcr-1.1*-producing *E. coli* from one patient (Patient 2) over two months. It is not clear what is the origin of these *mcr-1.1* isolates. We speculate that an *mcr-1.1*-producing *E. coli* strain was naturally present in the patient’s gut microbiota and, after selective pressure caused by the antimicrobials used over the long hospital stay, this same strain was responsible for the urinary infection. The urine infection site is suggestive of a gut microbiota source after colonization with *mcr-1*-positive *E. coli* due to previous infection episodes. We base this hypothesis on the fact that all three isolates were classified as ST410, and the all-against-all genome comparison shows that these three isolates are the most similar to one another compared to the other five isolates (Table 2 and Supplementary Table 1). The genomic similarities observed between these three strains are high (minimum value 99.93%), and this needs to take into account that genome assembly was only at the draft level (meaning that the similarities could be even closer to 100% if the genomes had been finished). The inferred phylogenetic tree shows that the isolates from patient P2 cluster together.

In our study we observed two different *mcr-1-*carrying contig types, which can be described by the plasmid marker found in these contigs: IncX4 (seven isolates) and IncHI2A (one isolate). Wang et al. (2018) found 13 different plasmids carrying *mcr-1* worldwide, and IncI2 and IncX4 were the dominant plasmid types found.

*mcr-1* has been sporadically described among human clinical isolates with multidrug resistance profiles (MDR; Fernandes et al., 2016; Aires et al., 2017; Conceição-Neto et al., 2017; Dalmolin et al., 2017, 2018; Rocha et al., 2017; Lorenzoni et al., 2018; Oliveira et al., 2018; Tada et al., 2018; Tonini et al., 2018; Farzana et al., 2019). However, the most frequently reported detection of *mcr-1* is in environmental and food-animal strains. In addition, the environmental strains appear to be more pathogenic than those recovered from human infections, including ESβL and carbapenemase-producing isolates (Fernandes et al., 2016, 2017; Monte et al., 2017; Drali et al., 2018; Tuo et al., 2018; Wu et al., 2018; Barlaam et al., 2019; Rumi et al., 2019; Yang et al., 2019; Dandachi et al., 2020; Loayza-Villa et al., 2020). In a global systematic review, Elbediwi et al. (2019) state that the estimated prevalence of *mcr-1* pathogenic *E. coli* was higher in food-animals than in humans and food products, which suggests a role for foodborne transmission. Brazil is a major producer and exporter of chicken, pig and poultry meat, and colistin sulfate, until recently, was used as a growth promoter (Morales et al., 2012). According to Fernandes et al. (2016) this can support a link between the use of colistin in livestock and the appearance of colistin resistance in *E. coli* isolates. Chiba et al. (2019) have reported *mcr-1* in chicken meat imported from Brazil. Yang et al. (2019) state that the multidrug resistance associated with livestock not only affects animal production, but also human health, by contaminating the food chain. However, the low *mcr-1* detection rate in human infections, observed in our study, allows us to hypothesize that this mechanism is not frequent inside a hospital environment, as observed with KPC-producing *K. pneumoniae* and OXA-producing *A. baumannii* strains. Additional investigation is required in order to determine the real origin and relevance of the polymyxins plasmid-mediated *mcr-1* mechanism.

We observed three different genomic contexts for the *mcr1.1-pap2* cassette in the genomes here presented. This diversity suggests a capacity of *mcr-1* to be mobile, using different cassette compositions, and to insert itself in variable contexts, as observed in other studies. Snesrud et al. (2016) state that, after transposition of the *mcr-1* cassette, fragments of the transposon may be lost. Furthermore, the *mcr-1* cassette may be inserted in various locations in the genome, including the chromosome; Wang et al. (2018) have presented similar results.

The presence of Insertion Sequences (ISs) in the *mcr-1* genomic context has been described in diverse studies, and Snesrud et al. (2016) proposed a model of transposition of the genomic cassette *mcr-1* – *pap2* flanked by two copies of IS*Apl1* in the same orientation, and suggest that the *mcr-1* cassette is mobilized as a composite transposon. These authors have also described a single copy of IS*Apl1* upstream of *mcr-1*. Different from observed in our study, IS*Apl1* is an IS30-family sequence. Furthermore, in isolate Ec721, the IS1-family transposase is inserted upstream of the *mcr-1* cassette, on the opposite strand, and may therefore not play a role in the cassette mobility. Wang et al. (2018) observed higher presence of ISApl1 transposon background from China isolates compared to other countries, and suggest a single mobilization of IS*Apl1*-*mcr-1* – *pap2* – IS*Apl1* fragment with subsequent diversification during global spread. However, these authors state that the origin of *mcr-1* prior to its mobilization remains unclear.

## Conclusion

Our results suggest that, in the setting and period investigated, intra-hospital *E. coli mcr-1.1*-carrying plasmid dissemination took place. The evidence are the six isolates for which we found the identical *mcr-1-pap2* cassette associated with a IncX4-plasmid, which were isolated from four different patients. The case of the Ec502 isolate, which is the most different from the others, both in the genomic sequence as a whole, as well as in the *mcr-1-pap2* cassette and plasmid incompatibility complex type, is more difficult to interpret with available data. It might have acquired its *mcr1.1* gene in the hospital, or it might have come from the outside already with the *mcr-1.1* gene, possibly as part of the patient’s microbiota. The genome sequencing of new isolates, coupled with detailed patients’ histories, may help clarify this issue. Such additional sequencing will also help with the much-needed continued surveillance for the *mcr-1.1* gene.

## Data Availability Statement

The datasets presented in this study can be found in online repositories. The names of the repository/repositories and accession number(s) can be found in Table 3.

## Author Contributions

RG contributed with study design, conduction of experiments, data analysis, and writing and revision of the manuscript. CP and JM participated in the conduction of experiments and data analysis. MM, AC, MF, FM, and NR contributed with conduction of experiments. JP and FR contributed with critical reading and supervision. AD contributed with funding and supervision. JS contributed with conduction of experiments, data analysis, writing, and revision of the manuscript. All authors contributed to the article and approved the submitted version.

## Conflict of Interest

The authors declare that the research was conducted in the absence of any commercial or financial relationships that could be construed as a potential conflict of interest.
