# Supplementary material for: Genomic Characterization of mcr-1.1-Producing Escherichia coli Recovered From Human Infections in São Paulo, Brazil
Source: Front Microbiol. 2021 Jun 9;12:663414. doi: 10.3389/fmicb.2021.663414 (PMC8221240; doi:10.3389/fmicb.2021.663414)
Supplement: Supplementary file 1 [file Table_1.DOCX]

**Supplementary Table S1.** FastANI similarities between the eight genomes that were sequenced (%).

|  | **Ec1057** | **Ec1177** | **Ec482** | **Ec483** | **Ec502** | **Ec716** | **Ec721** | **EcHC891** |
| --- | --- | --- | --- | --- | --- | --- | --- | --- |
| **Ec1057** | **100** | 99,5226 | 98,4519 | 98,4334 | 96,9809 | 98,4959 | 99,3661 | 98,2176 |
| **Ec1177** | 99,5691 | **100** | 98,436 | 98,4424 | 97,0542 | 98,4252 | 99,3088 | 98,2544 |
| **Ec482** | 98,4578 | 98,445 | **100** | 99,9732 | 96,9702 | 99,9573 | 98,3244 | 98,6353 |
| **Ec483** | 98,4538 | 98,4263 | 99,9745 | **100** | 96,9565 | 99,9474 | 98,3331 | 98,6966 |
| **Ec502** | 97,107 | 97,1195 | 96,9953 | 96,9971 | **100** | 97,0782 | 96,956 | 97,0724 |
| **Ec716** | 98,458 | 98,3952 | 99,9499 | 99,9327 | 97,0254 | **100** | 98,331 | 98,6506 |
| **Ec721** | 99,3804 | 99,3463 | 98,2884 | 98,3406 | 97,0945 | 98,4035 | **100** | 98,1676 |
| **EcHC891** | 98,2322 | 98,2859 | 98,6963 | 98,7136 | 97,038 | 98,7423 | 98,1571 | **100** |

Highlighted in yellow are the three strains isolated from the same patient.

Highlighted in gray is the only strain with Genomic Context III for gene *mcr1-1*.
