# Supplementary material for: Genomic Characterization of mcr-1.1-Producing Escherichia coli Recovered From Human Infections in São Paulo, Brazil
Source: Front Microbiol. 2021 Jun 9;12:663414. doi: 10.3389/fmicb.2021.663414 (PMC8221240; doi:10.3389/fmicb.2021.663414)
Supplement: Supplementary file 2 [file Table_2.DOCX]

| Isolate | *mcr-1*^1^ | Minimal Inhibition Concentration (µg/mL)^2^ | | | | | | | | | | | | | |
| --- | --- | --- | --- | --- | --- | --- | --- | --- | --- | --- | --- | --- | --- | --- | --- |
|  |  | COL | AMP | PPT | CFU | FOX | CAZ | AXO | CFP | ERT | IMI | MER | AMI | GEN | CIP |
| EcHC891 | POS | 4 | >32 | <=4 | 4 | 16 | <=1 | <=1 | <=1 | <=0,5 | <=0,25 | <=0,25 | <=2 | 2 | >4 |
| Ec482 | POS | 8 | >32 | >128 | 16 | 32 | <=1 | <=1 | <=1 | <=0,5 | <=0,25 | <=0,25 | <=2 | >16 | >4 |
| Ec483 | POS | 8 | >32 | 32 | 16 | 32 | <=1 | <=1 | <=1 | <=0,5 | <=0,25 | <=0,25 | <=2 | >16 | >4 |
| Ec502 | POS | 8 | >32 | <=4 | 4 | <=4 | <=1 | <=1 | <=1 | <=0,5 | <=0,25 | <=0,25 | <=2 | <=1 | <=0,25 |
| Ec507 | NEG | >16 | >32 | 64 | 4 | <=4 | <=1 | <=1 | <=1 | <=0,5 | <=0,25 | <=0,25 | <=2 | <=1 | <=0,25 |
| Ec509 | NEG | >16 | >32 | <=4 | >64 | 32 | 2 | >64 | 32 | 4 | <=0,25 | 1 | <=2 | <=1 | <=0,25 |
| Ec516 | NEG | >16 | >32 | <=4 | >64 | <=4 | 4 | 32 | <=1 | <=0,5 | <=0,25 | <=0,25 | <=2 | <=1 | >4 |
| Ec609 | NEG | >16 | <=2 | 2 | 2 | <=4 | <=1 | <=1 | <=1 | <=0,5 | <=0,25 | <=0,25 | <=2 | <=1 | <=0,25 |
| Ec716 | POS | 4 | >32 | 64 | 4 | 8 | <=1 | <=1 | <=1 | <=0,5 | <=0,25 | <=0,25 | <=2 | >16 | >4 |
| Ec721 | POS | 4 | >32 | <=4 | 2 | <=4 | <=1 | <=1 | <=1 | <=0,5 | <=0,25 | <=0,25 | <=2 | <=1 | <=0,25 |
| Ec864 | NEG | >16 | 8 | <=4 | 4 | <=4 | <=1 | <=1 | <=1 | <=0,5 | <=0,25 | <=0,25 | <=2 | <=1 | <=0,25 |
| Ec891 | NEG | >16 | >32 | >128 | >64 | >64 | >64 | >64 | >64 | >8 | >16 | >16 | >64 | >16 | >4 |
| Ec904 | NEG | >16 | >32 | 32 | 4 | <=4 | <=1 | <=1 | <=1 | <=0,5 | <=0,25 | <=0,25 | 4 | 2 | <=0,25 |
| Ec959 | NEG | >16 | >32 | <=4 | <=1 | <=4 | <=1 | <=1 | <=1 | <=0,5 | <=0,25 | <0,25 | <=2 | <=1 | >4 |
| Ec978 | NEG | >16 | <=2 | <=4 | 2 | <=4 | <=1 | <=1 | <=1 | <=0,5 | <=0,25 | <=0,25 | <=2 | <=1 | <=0,25 |
| Ec1057 | POS | 8 | >32 | <=4 | 4 | <=4 | <=1 | <=1 | <=1 | <=0,5 | <=0,25 | <=0,25 | <=2 | >16 | <=0,25 |
| Ec1087 | NEG | >16 | <=2 | <=4 | 4 | <=4 | <=1 | <=1 | <=1 | <=0,5 | <=0,25 | <=0,25 | <=2 | <=1 | <=0,25 |
| Ec1135 | NEG | 32 | <=2 | <=4 | 4 | <=4 | <=1 | <=1 | <=1 | <=0,5 | <=0,25 | <=0,25 | <=2 | <=1 | <=0,25 |
| Ec1177 | POS | 4 | >32 | <=4 | 4 | <=4 | <=1 | <=1 | <=1 | <=0,5 | <=0,25 | <=0,25 | <=2 | <=1 | >4 |

**Supplementary Table S2.** Antimicrobial resistance profile of colistin resistant *E. coli* isolated between 2016 and 2017.

^1^POS: positive; NEG: negative.

^2^ The susceptibility category was interpreted according to CLSI (2020) breakpoints. Highlighted in dark grey are the resistant category. Highlighted in light grey are the intermediate category. No highlighted are the susceptible category. COL: colistin; AMP: ampicillin; PPT: piperacillin-tazobactam; CFU: cefuroxime; FOX: cefoxitin; CAZ: ceftazidime; AXO: ceftriaxone; CFP: cefepime; ERT: ertapenem; IMI: imipenem; MER: meropenem; AMI: amikacin; GEN: gentamycin; CIP: ciprofloxacin.
