# Supplementary material for: Genomic Characterization of mcr-1.1-Producing Escherichia coli Recovered From Human Infections in São Paulo, Brazil
Source: Front Microbiol. 2021 Jun 9;12:663414. doi: 10.3389/fmicb.2021.663414 (PMC8221240; doi:10.3389/fmicb.2021.663414)

**Supplementary File S3.** Antimicrobial resistance rates of colistin resistant *K. pneumoniae* isolated between 2016 and 2017, by using Vitek-2 automated method.


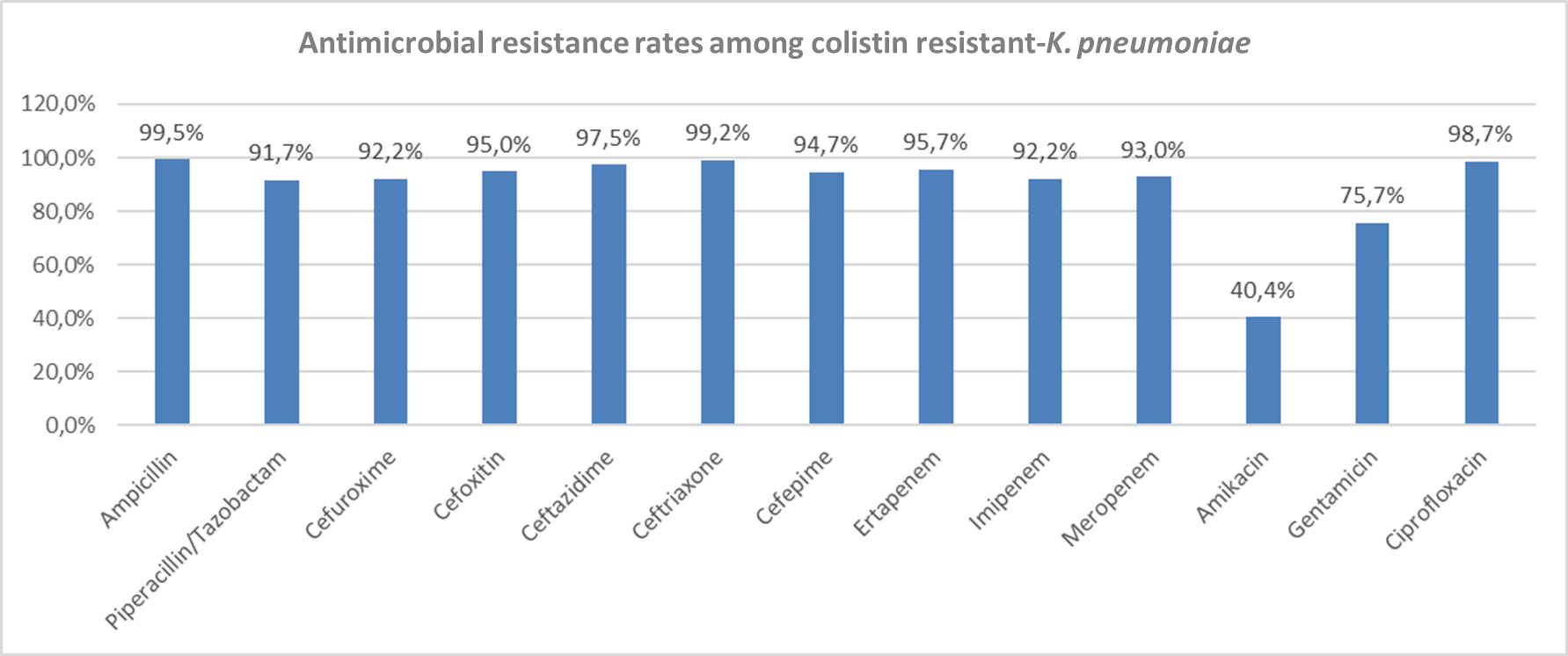

Supplement: Supplementary file 3 [file Table_3.DOCX]
